# Patients’ Experiences of Using a Consumer mHealth App for Self-Management of Heart Failure: Mixed-Methods Study

**DOI:** 10.2196/13009

**Published:** 2019-05-02

**Authors:** Leanna Sarah Woods, Jed Duff, Erin Roehrer, Kim Walker, Elizabeth Cummings

**Affiliations:** 1 School of Nursing University of Tasmania Darlinghurst Australia; 2 St Vincent's Private Hospital Sydney Darlinghurst Australia; 3 School of Nursing and Midwifery University of Newcastle Newcastle Australia; 4 School of Technology, Environments and Design University of Tasmania Hobart Australia; 5 School of Health Information Science University of Victoria Victoria, BC Canada

**Keywords:** heart failure, mobile health (mHealth), mobile apps, usability study, Mobile Application Rating Scale, patient experience, self-management, mobile phone

## Abstract

**Background:**

To support the self-management of heart failure, a team of hospital clinicians, patients, and family caregivers have co-designed the consumer mobile health app, *Care4myHeart*.

**Objective:**

This research aimed to determine patient experiences of using the app to self-manage heart failure.

**Methods:**

Patients with heart failure used the app for 14 days on their own smart device in a home setting, following which a mixed-methods evaluation was performed. Eight patients were recruited, of whom six completed the Mobile Application Rating Scale and attended an interview.

**Results:**

The overall app quality score was “acceptable” with 3.53 of 5 points, with the aesthetics (3.83/5) and information (3.78/5) subscales scoring the highest. The lowest mean score was in the app-specific subscale representing the perceived impact on health behavior change (2.53/5). Frequently used features were weight and fluid restriction tracking, with graphical representation of data particularly beneficial for improved self-awareness and ongoing learning. The use of technology for self-management will fundamentally differ from current practices and require a change in daily routines. However, app use was correlated with potential utility for daily management of illness with benefits of accurate recording and review of personal health data and as a communication tool for doctors to assist with care planning, as all medical information is available in one place. Technical considerations included participants’ attitudes toward technology, functionality and data entry issues, and relatively minor suggested changes.

**Conclusions:**

The findings from this usability study suggest that a significant barrier to adoption is the lack of integration of technology into everyday life in the context of already established disease self-management routines. Future studies should explore the barriers to adoption and sustainability of consumer mobile health interventions for chronic conditions, particularly whether introducing such apps is more beneficial at the commencement of a self-management regimen.

## Introduction

Heart failure affects at least 26 million people worldwide [1], including more than 1 million Australians [2], and its prevalence is expected to rise [1]. This complex, highly symptomatic syndrome is associated with high health care costs, high readmission rates, and poor clinical outcomes [3]. Targets to improve functional outcomes, psychosocial outcomes, burden of care, and survival of patients with heart failure have resulted in a call for safe, person-centered, evidence-based action [3]. It is especially necessary to ensure equity of care for all patients through the efficient use of resources as well as support to empower patients and caregivers in long-term care [4].

Self-management support, specifically for nonpharmacological requirements, is critical to the effective management of heart failure [2] and is often delivered through educational measures [3,5,6]. Appropriate self-management of heart failure involves daily weight monitoring, fluid restriction, dietary modifications, and exercise alongside regular monitoring and follow-up [2]. In the home setting, recording and recognizing changes such as increased weight, fluid retention, and worsening symptoms, which are indicative of worsening heart failure, can allow patients to get help early [6]. However, challenges with translating guidelines into practice put patients at risk of suboptimal care [2], with the complexity of self-management of heart failure contributing to poor adherence [7].

Rapid improvements in computing capability paired with the popularity of mobile phones in our communities provide more opportunities in health care delivery [7]. Due to this potential, mobile health (mHealth) interventions for heart failure continue to expand; however, this expansion is accompanied by challenges in technology adoption. Reliability of equipment [8], limited technical support [8], cognitive impairment [9], and variable interest in self-recording of health measurements [9] are a few factors affecting use in this patient population. Older people, who have a prevalence of heart failure three times greater than that of the general population [10], have variable levels of willingness to adopt technology [9]. They may lack confidence in their knowledge of heart failure and rely on informal and formal caregivers for guidance [9]. Perceived usefulness and ease of use are considered the most important factors for mHealth adoption [11]. This poses specific challenges when designing interventions aimed to engage patients in self-management of heart failure and highlights the importance of using patient perceptions in newly developed interventions. Further, in a recent review, of the 34 consumer apps targeting heart failure on the commercial app stores, only 3 were evaluated in peer-reviewed articles [12], indicating the importance of disseminating research findings to advance consumer mHealth.

This study is part of a larger research program where *Care4myHeart*, an mHealth app for self-management of heart failure was developed in our hospital by a team of clinicians, patients, and family caregivers. The diverse group of stakeholders collaborated to design an app that was relevant and useful to target users and consistent with the evidence-based heart failure guidelines. The aim of this paper was to explore patients’ experiences of and feedback after using the app.

Specific research questions were as follows:

1. What were the patients’ experiences of using the *Care4myHeart* app?
2. What is the perceived impact of the app on self-management of heart failure?

## Methods

A 14-day usability study was performed using a mixed-methods evaluation to determine patient experiences of using the mHealth app for self-management of heart failure.

### Participants

Self-selecting participants were recruited from cardiac inpatient units at a metropolitan private hospital in Sydney, Australia, via posters and flyers located in common patient areas. Medical and nursing staff members were informed of the research and referred patients who voiced their interest in participating. We included English-speaking individuals with heart failure who were not highly dependent on medical care, resided at home, were able to provide feedback, and owned a smart device capable of housing the app. Participants were excluded if they were involved in the co-design of the app, were cognitively impaired, or were otherwise unable to use the app. We aimed for a sample size of 8-10 participants, because up to 80% of usability problems can be identified by this number of users [13].

### Intervention

**Figure 1:**
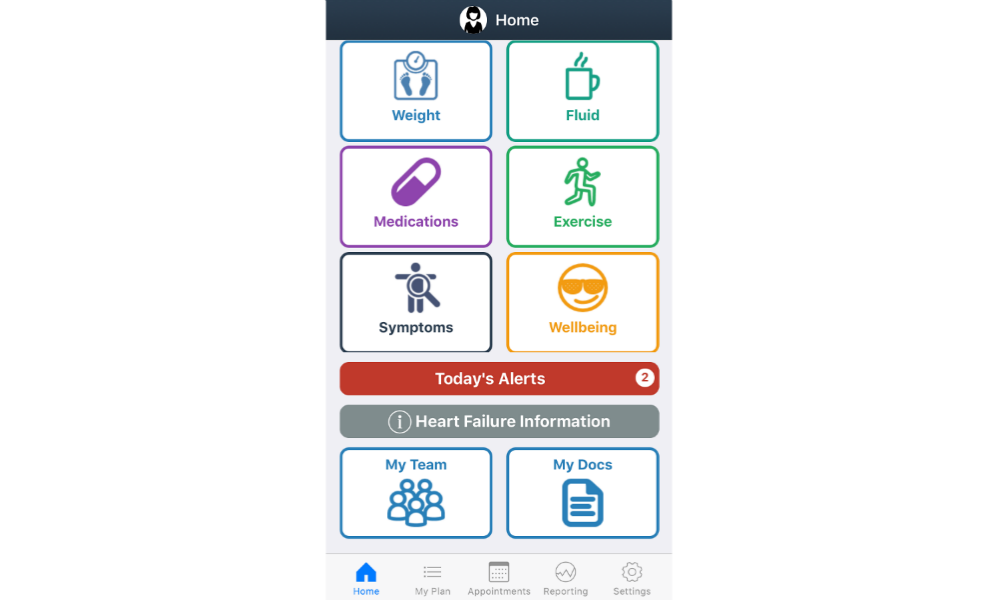
Sample home screen.

**Figure 2:**
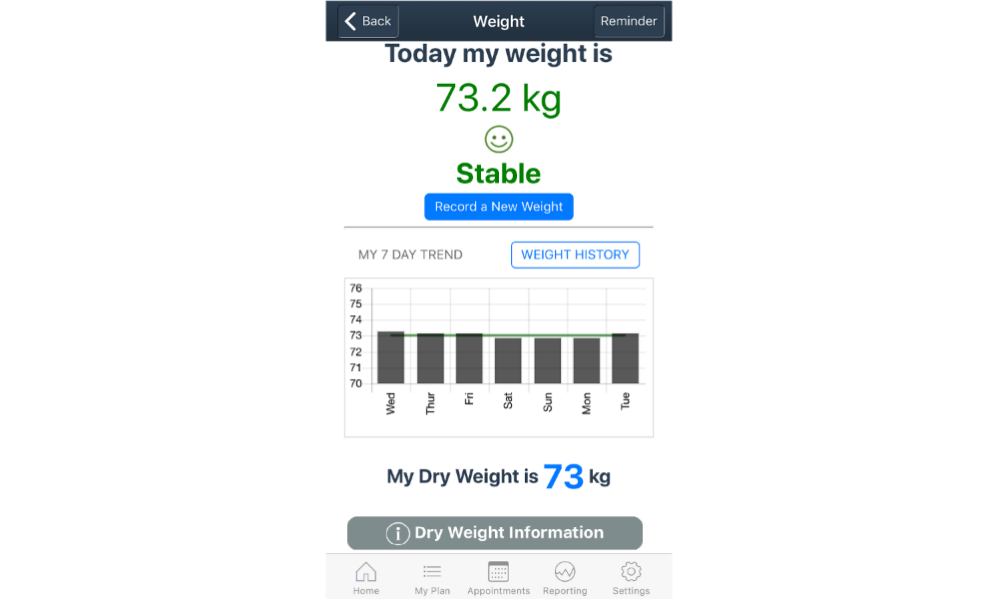
Sample weight screen.

**Figure 3:**
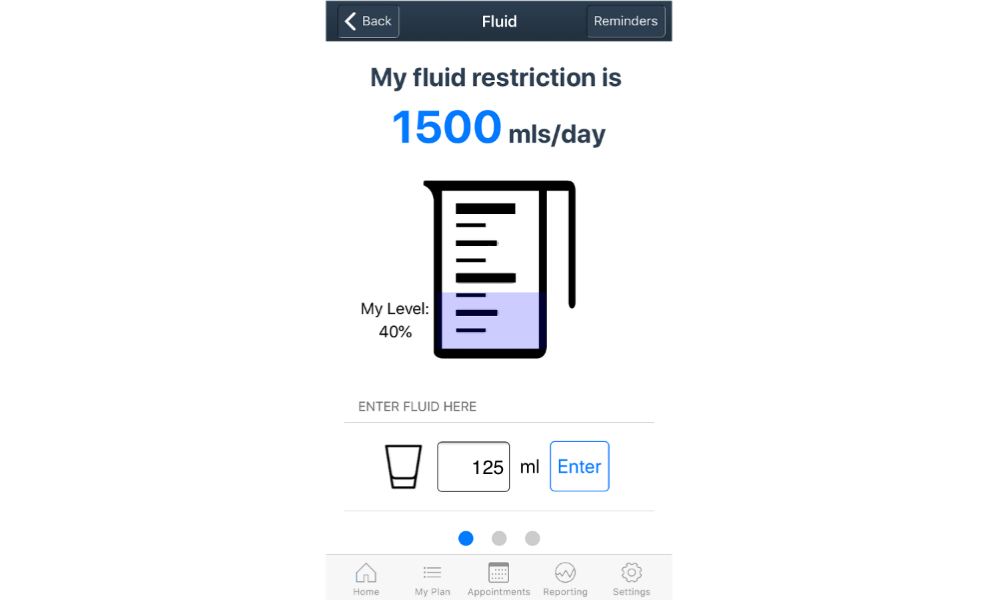
Sample fluid restriction screen.

Details of the co-design process of the mHealth app are reported elsewhere [14-17]. The final design of the self-management app has three main sections: *Home screen*, *My Plan*, and *Health Management*. The *Home screen* provides a shortcut to the priority *My Plan* icons based on patient goals, and a reminder summary. The *My Plan* section includes nine important components of self-management of heart failure: medications, symptoms, exercise, weight, fluid, well-being, diet, blood pressure and pulse, and future plans. A *Health Management* section contains a medical documentation repository, appointment calendar, and health care professional contact details. The app provides the opportunity to collect, track, and evaluate patient-entered data. Reminders, alerts, infographics, videos, health professional advice, and information pages throughout the app aim to guide patients to manage their heart failure. Sample user interfaces demonstrating the home, weight, and fluid restriction screens are presented in Figures 1, 2, and 3, respectively.

### Study Procedures

The *Care4myHeart* app was downloaded to patients’ own iOS or android smartphone or tablet device after procedures were explained and patient consent was obtained. A researcher spent 10-30 minutes providing an overview of the app interface, assisted with completing the personalized settings (dry weight, daily fluid restriction volume, daily step count aim, physical activity goals, and reminders), and determined self-management priorities based on patient preferences. Participants were asked to use the app as frequently as required to assess its usability, aiming for at least daily use over a 2-week period. Participants were encouraged to contact the research team by phone or email if they encountered problems or had questions throughout the study. For quality and safety reasons, participants were instructed to continue with their regular care regime in collaboration with their health care providers. Ethical approval for this study was obtained from the University of Tasmania and St Vincent’s Private Hospital Sydney.

### Data Collection

As soon as practically manageable after the completion of a 14-day period, participants reported their experience of using both qualitative and quantitative methods.

First, participants were asked to complete the Mobile Application Rating Scale (MARS) [18] either electronically (sent via email) or on paper (sent by post or completed in person during the interview). The 23-item MARS is a multidimensional measure of the four objective app quality indicators: engagement, functionality, esthetics, and information (which together form the overall app quality score). In addition, it includes a subjective quality subscale [18]. As *Care4myHeart* was not available in the app stores during the time of the study, we modified the MARS to 19-items, excluding four items because they were not applicable: accuracy of app description (item 13), goals (item 14), credibility (item 18), and evidence base (item 19). These items were removed from the mean score calculation as per the guidelines [18]. A supplementary, modifiable “app-specific” section assessed the perceived impact of the app on users’ target health behaviors [18], in this case, improved heart failure self-management. MARS items are scored on a 5-point Likert scale (1=inadequate, 2=poor, 3=acceptable, 4=good, and 5=excellent) [18]. The version used for this study is provided in Multimedia Appendix 1.

Second, participants were asked to attend an interview on the hospital campus or via phone, depending on patients’ preference. A semistructured interview schedule included questions such as “What worked well and what could be improved?” “What functions did you use and why?” and “Would this application impact the way you look after your health?” Participants were given the opportunity to share experiences, communicate thoughts, and voice perspectives through open-ended and probing questions. App use was self-reported by participants themselves, as no usage data were collected in this study. Data were collected in June and July 2018.

### Data Analysis

Data were de-identified and treated confidentially. MARS data were managed in the database software program Excel (Microsoft Corp, Redmond, WA), with mean scores produced by calculation of participant subscale scores. Interviews were transcribed verbatim and thematically analyzed using Braun and Clarke’s process [19]. The process involved familiarization of the data through re-reading transcripts (Step 1), generation of initial codes and writing them directly on the transcript segments considered interesting or meaningful to the analyst (Step 2), organization of codes into potential themes (Step 3), review of themes through checking and generating a thematic “map” (Step 4), generation of clear definitions and names for each theme (Step 5), and production of the report with compelling examples through a final analysis (Step 6) [19]. Data analysis in Steps 1 and 2 was conducted by the lead author (LW). Steps 3 to 6 were performed visually and collaboratively, with the themes confirmed by group discussion with the coauthors.

## Results

### Participant Characteristics

Eight participants consented and commenced the usability study. All participants were male (n=8), most lived with a spouse/partner (n=7) and were currently employed (n=5), and more than half resided in a rural location outside the metropolitan area (n=5). The average age of participants was 69 years (range: 61-84 years).

One participant discontinued the study after reporting technical challenges with a software update that occurred during the 14-day period. A second participant died prior to the final interview and collection of the MARS. Six of the eight participants completed the study with the survey and interview. The interview length ranged from 18 to 29 minutes.

### Mobile Application Rating Scale App Quality Scores

Table 1 presents the four subscale scores (engagement, functionality, esthetics, and information), which make up the overall quality score, as well as the subjective quality score (representing satisfaction) and app-specific score (representing behavior change).

The overall app quality score was 3.53 of 5. Of the four subscales, the highest scores were for esthetics (3.83) and information (3.78), followed by engagement (3.37) and functionality (3.33); all scores were above the minimum acceptability score of 3.0. The highest-scoring individual items were layout (4.17), visual information (4.17), interest (3.83), and quality of information (3.80). The lowest scores per item were for performance (2.67), customization (3.00), and interactivity (3.00).

The subjective quality subscale representing app satisfaction scores showed an average of 3.29 of 5. Most participants would use the app more than 50 times in a 12-month period (n=7) and recommend the app to people who might benefit from it (n=4), but would not pay for the app (n=4). The mean star rating, comparable to the star rating on the app stores, was 3.33.

The lowest mean score was in the app-specific section representing the perceived impact of using the app on health behavior change (2.53). The app may have some impact on increased awareness regarding self-management of heart failure (3.17) but was rated “poor” on the perceived impact of the app on attitude, intention to change, help seeking, and overall behavior change (2.33).

### Interview Findings

Analysis of interview transcripts resulted in 3 themes and 10 subthemes (Textbox 1).

#### Theme 1: App Use

Most participants used an android device (smartphones: n=2, tablets: n=2) and two used iPhones. Five participants had both a smartphone and a tablet device. Tablets were kept at home, and smartphones were not necessarily used for internet access. However, those who carry their smartphone in their pocket saw the benefit in data entry throughout the day. iOS users spoke about using their device with greater understanding and confidence than Android users in our sample; the former were also the two youngest participants. Patients self-reported app use for an average of 5-10 minutes once or twice a day on most days during the usability study. The app was used independently without family member involvement. Usage over the 14-day period decreased once users determined what was useful; however, version updates improved technical issues, with usage reportedly increasing after the updates.

##### Weight, Fluid Restriction, and Step Counter

The weight and fluid restriction sections were most frequently used. The quick speed of recording weight and weight alerts was highlighted as positive features. One participant described how beneficial the fluid recorder was:

The most beneficial feature for me at this point in time is the fluid intake...the fluid counter is excellent. I love it, absolutely love it.

P8

Fluid volumes were entered either throughout the day or at the end of the day in the fluid restriction section of the app:

I wouldn’t put in fluid every time I had 100ml of fluid - I put it all in at the end of the day.

P7

Some found the app more convenient for self-management of fluid restriction than traditional means of recording fluid volumes because it was portable:

Beforehand what I was doing I had a measuring cup...I think the app is more friendly for me to use...I’ve got that in my pocket, I can always - when I’m out and about - I can make an input on my smartphone and it’s just so convenient.

P8

To a lesser extent, the step counter within the exercise section was used.

##### Use of Features

Not all features of the app were used by participants. Participants did not regularly use the symptoms, documents, medication list, and calendar sections, but many saw potential advantages in using these additional features stating, “I didn’t use everything but I can see other people could find it very useful” (P1). For example, due to the high frequency of medication changes in patients with heart failure, keeping an updated medication list was perceived as a positive feature. Participants did not use these features during the usability study stating that they “didn’t really get a chance to go through it” (P6), and “ah, I had a look but I didn’t use any of it functionally” (P7).

**Table 1:** Mobile Application Rating Scale subscale scores.

Subscale^a^ and item		Mean (SD)
**Engagement**		
	Entertainment	3.33 (1.03)
	Interest	3.83 (0.75)
	Customization	3.00 (0.89)
	Interactivity	3.00 (0.89)
	Target group	3.67 (0.82)
	Subscale mean	3.37 (0.69)
**Functionality**		
	Performance	2.67 (1.63)
	Ease of use	3.67 (0.52)
	Navigation	3.67 (1.03)
	Gestural design	3.33 (0.82)
	Subscale mean	3.33 (0.66)
**Esthetics**		
	Layout	4.17 (0.75)
	Graphics	3.67 (1.03)
	Visual appeal	3.67 (0.82)
	Subscale mean	3.83 (0.81)
**Information^b^**		
^ ^	Quality of information	3.80 (0.84)
	Quantity of information	3.60 (1.52)
	Visual information	4.17 (0.41)
	Subscale mean	3.78 (0.81)
Overall quality		3.53 (0.63)
**Subjective score**		
	Recommendation	3.50 (1.22)
	Use in 12 months	4.67 (0.82)
	Pay for the app	1.67 (1.03)
	Star rating	3.33 (0.82)
	Subscale mean	3.29 (0.70)
**App-specific items**		
	Awareness	3.17 (1.17)
	Knowledge	2.67 (0.52)
	Attitudes	2.33 (0.82)
	Intention to change	2.33 (0.82)
	Help seeking	2.33 (0.82)
	Behavior change	2.33 (0.82)
	Subscale mean	2.53 (0.71)

^a^Mobile Application Rating Scale values range from 1=inadequate to 5=excellent.

^b^The information quality score excluded items 13, 14, 18, and 19 from the Mobile Application Rating Scale.

**Summary of the themes and subthemes from participant interviews.**

- App use
  - Weight, fluid restriction, and step counter
  - Use of features
  - Graphs as visual representation of patient data

- Capacity for self-management
  - Established understanding of heart failure and self-management practices
  - App for daily management of illness
  - App as communication tool

- Technical considerations
  - Attitudes toward technology
  - Functionality
  - Data entry
  - Suggested changes

Participants did not watch the instructional exercises videos due to disinterest, personal preference to undertake their own form of exercise, and awareness that they would not continue after a few weeks of watching the same videos. Additional reasons for not using all the features of the app included technical issues and a lack of perceived value for the time required for data entry. One participant commented on why he did not take the time to enter his medications and doctor’s contact details into the app:

I’m just trying to wait until I get my medications stabilised before I make the inputs...My doctor’s names and all of that information I haven’t put that in yet but I will over time. It’s just – ah – I’ve I tell you I’ve been so busy since getting back [home after hospital], just busy busy busy and relaxing after 4 weeks in the hospital.

P8

Heart failure information was considered useful for a few patients; however, most participants felt the information was already known to them; one said, “there’s no new material for me actually” (P6). Another participant explained how the lack of new information relates to perceived utility of the app:

For me it’s things I already know...I know I’m big on diet, big on health, so a lot of this information in the app I already know but it just reinforces it...I do enjoy the app but I don’t need it.

P8

##### Graphs as a Visual Representation of Patient Data

Visual representation of patient data through graphs was a positive feature of the app, specifically for self-awareness. For daily weight management, graphs were deemed useful, accurate, and relevant and provided feedback to users, as viewing 7-day weight trends heightened self-awareness. A participant explained how the weight trend allowed him to be more “weight aware” (P2), and another appreciated the visual representation of health data specifically:

In a graphical sense you see [the weight trend] straight away. And your brain functions on that rather than on just a list of numbers.

P7

Self-awareness regarding mobility was deemed beneficial in the exercise section as well. The 7-day step counter graph provided an accurate picture of the mobility status to patients who used the feature:

I’m just trying to keep track of how much activity I’m doing, to make sure I’m…keeping moving.

P1

Graphical representation of patient data provided learning opportunities. Monitoring the link between fluid intake and fluid congestion can be challenging. However, graphing these data may assist to review previous day’s fluid intake and to cross reference this information with fluid congestion symptoms, which may be caused by previous days’ nonadherence:

[It] appears in your record that you can go back and look and then gives you some sort of positive understanding about what you might have done wrong...your ankles swell up the following morning and you think “ahhhh dopey bugger, I should have bloody been more careful” so and they’re lessons we all learn...recognising [I’ve] gone over [my fluid restriction].

P7

#### Theme 2: Capacity for Self-Management

Participants were unsure how *Care4myHeart* would fit into the way they currently *understand* heart failure and conduct self-management, as using the app for heart failure would require a fundamental change in routine. However, there was potential benefit to heart failure self-management for *daily management of illness* with the benefits of accurately recording and reviewing personal health data, and as a *communication tool* for doctors to assist with care planning, as all medical information is in available one place. These three subthemes are discussed below.

##### Established Understanding of Heart Failure and Self-Management Practices

Participants found their own way to self-manage their health. Living with the condition for many years, understanding the importance of self-management, and setting goals regarding self-management had contributed to their existing behaviors embedded into daily life. There were many existing self-management strategies: use of a measuring jug on the kitchen bench for fluid intake monitoring, digital calendars, shared household calendar on the back of the pantry door for medical appointments/reminders, liaising with specialist nurses via email, and paper files containing medical documentation.

Participants reported satisfaction with their current health care. Notably, patients reported easy access to health care professionals for regular follow-up, ongoing education/information, and question answering. Participants spoke highly of their current general practitioner, cardiologist, and heart failure nurses:

I’ve got the heart nurse’s phone number and mobile number too. She’s absolutely fantastic.

P3

Participants were aware of and followed a self-management care plan in conjunction with their health care team, knowing their condition is life-limiting. Satisfaction with these current routines was demonstrated:

I mean why do I need an app to tell me that ah “do this, do this and this, and you’re going to have a better life”? Whereas I get all of this so-called experts, the doctors and all of the information they give you, they tell you the same thing [as the app]...I don’t necessarily need an app. Personally, I’m going to do the right thing because I want to live...I know I’m dying. I’m dying as we speak, there’s no secrets here but I want to live so I’m going to do the right things.

P8

Existing self-management strategies were in a different location or format from the app. Participants compared the convenience of their existing strategies to using the app for self-management. Particularly, participants critiqued the need to “go to various pages on the program” (P3) to view health data, as participants commonly documented information in a notebook or electronic spreadsheet. These existing records have been tailored to the specific requirements considered important by the patients themselves or their health care team. The benefit of these existing daily records was the ability to view their health status at a glance and as a self-management checklist:

I can just look at one page and get the whole picture of what’s happening...it’s all on one page, so I can tick something when I’ve taken it...I just have a look at [the page] and see that I’ve done everything that day and basically...well that’s the day done, I’m complete.

P3

Further, existing strategies were considered easy and time efficient in everyday life, as one participant explained about maintaining his fluid restriction throughout the day using other strategies compared with using the app:

I would personally keep going the way I’m going cos of the ease of doing it...[T]he easy things I’d rather just do easy, like the water in the jug...where the app’s stuck in my bedroom most of the time. I’ve gotta go and turn it on, I’ve gotta go bang, bang, bang, and by the time I’ve sorta done the water in the jug I’ve well and truly finished before probably I’ve even get into the program properly.

P3

Although the app may assist in monitoring specific self-management activities like weight or fluid intake, it did not seem to embody the complexity of self-management of heart failure. Participants communicated a good understanding of heart failure (with the exception of one participant who was not familiar with the term “dry weight”). They correctly understood that fluid congestion was variable, fluid intake and diuretic medications are directly linked to fluid status, and regular self-assessment for abdominal/ankle edema was necessary. Understanding these concepts of heart failure involved a more thorough and subjective self-assessment, which was not directly equivalent to the setting’s parameters within the design of the app. One participant explained his thought process while conducting a self-assessment, which was a more complex process than simply adhering to a daily fluid restriction:

Sometimes I will go over my fluid intake which is 1.2 [litres], sometimes I go over because I’m looking at the way I feel...I’m doing a couple of things. I’m looking at the fluid intake but I’m also looking at my body or seeing the way I feel...I’m looking at how dry I am…I’ll just drink a little bit more and not get a doctor review [because] I haven’t started to pick up any signs of oedema.

P8

##### App for Daily Management of Illness

The app provided a routine to manage health data like weight. Participants explained that “it generates a discipline to maintain the information” (P2) specifically regarding “the daily management of my fluid balance, it takes a lot of adjustment...to get the balance right” (P1). Entering weight was quicker using the app than the usual format of documenting weight for some proclaiming “this is a quicker way of doing it, like most computers it can store information well” (P2).

Recording health information within the app on a daily basis was considered more accurate than manual measures or memory. One participant explained how he normally relies on memory:

I don’t record it as such but I check it every couple of days keeping a mental note – I just want to make sure there are no big variations from day to day so that’s all I look for [but with the app] it’s nice to have that trend, I like it, it gives you a more accurate picture.

P6

The health data repository and feedback within the app provided an opportunity to view a person’s health status more objectively. For example, accurate recording of health data might help family members seek care appropriately during times of worsening heart failure:

If you go into denial stage and don’t pay attention to the weight because you don’t want to go into hospital or something, now they can look and see “Ah well that’s not right – we should get you to the doctor” so I think it would help.

P1

##### App as Communication Tool

The app was considered a potential tool to communicate with doctors and other health professionals about assisting with care planning. Participants explained how the app could facilitate accurate information sharing:

[The app]enables you to communicate with your medical practitioner in a fairly accurate - one would hope - way, about what’s been going on and therefore one would hope, if you were the medical practitioner, I suppose it would cause the medical practitioner a better basis of making decisions about your medical care.

P2

As a potential communication tool, the app could assist doctors with patient assessment. Participants frequently spoke of the potential to show doctors the graphs representing health-related trends of recent days in a consultation, as “it’s quick” (P7), or over the phone, as “If you had it on a phone you could just say [to the doctor] ‘Look, I’ll send this through to you”’ (P7). Another participant agreed with this potential:

The concept is good because you can take your tablet along to your doctor and he says “well how have you been?” and you can say “well there you are, there’s my weight, there’s my blood pressure,” so you’ve got that information available.

P2

Having medical information in one place was deemed useful if all relevant data were stored in the app. Digital storage of personal medical records was considered “very powerful and very useful” (P7), as participants saw benefit in having “everything in one place” (P6) and “recorded accurately” (P1). Digital copies of medical information were considered “much easier rather than carrying an actual physical document. Sometimes I forget to take it” (P6). The potential to use the app as a communication tool was deemed especially valuable for new or temporary doctors and during medical emergencies:

Just air drop [my current medication list] from your phone to the doctor in casualty or whatever I think’s a great, very good idea...I think that would be helpful for a lot of people especially if you come into hospital somewhere hypoxic...unconscious or whatever...or too breathless to talk about it. I’ve got a very very extensive list of drugs that I’m on, I think it’s 35 tablets a day usually, so having that list when I’ve gotta provide it, makes it much easier.

P1

However, no participants reported using the app with members in their health care team during the time of the study. Further, the version used for the usability study was not set up for third-party access.

#### Theme 3: Technical Considerations

There were technical considerations influencing the experience of using the app, including *attitudes toward technology* and *functionality* and *data entry* issues. These subthemes are reported in the following section alongside the final subtheme—numerous *suggested changes* —to improve the app’s design.

##### Attitudes Toward Technology

Predominantly, participants were not regular users of smart devices for apps or health. Three sample quotes demonstrated minimal interest in using smart devices overall:

I’m not a big user of phones, especially mobile phones.

P8

I don’t particularly like turning computers on anyhow, I mean I’d go a fortnight without reading my emails.

P3

I’m a dinosaur and not used to using texting.

P7

Trust was one reason a participant would not use internet banking or purchase products using a credit card (P3). Participants reported using their smart devices for Google calendar, checking the weather forecast, playing games (CandyCrush, solitaire, or crosswords), and internet searches, and only a few used emails. In relation to technology use for health, one participant reported using a health app for self-management of heart failure and another stored his current medication list in the notes section of his smartphone. No participants reported storing medical documents electronically.

Participants believed in the inevitable advancement of technology in the contemporary era, and this was perceived to include the acceptance of health apps like *Care4myHeart* for younger generations. With the everyday use of smartphones, the younger generation “would approach it completely differently” (P7). Another participant explained:

I think for really the next generation and computer nerds at the moment you’re on a winner there, I really do...As you get the younger ones come through you’ll be fine, which will happen just over time.

P3

Attitudes toward technology by family members appeared consistent with those of the participants. There were no reports of receiving assistance from family members by using the app:

[My wife is] less techno-cradic [sic] than I am. I mean she went from a phone with a touchscreen back to a phone with push buttons on it, that’s what she likes.

P7

The personal nature of smartphones may impact the divide between family members:

[It is] my phone so she didn’t really take a closer look.

P8

##### Functionality

Technical challenges were reported to affect usage, which was more prevalent in Android than iOS devices. Issues with downloading the version update on Android caused one participant to discontinue participation in the study. A second participant was unable to download the updated Android version but managed to continue with the original version downloaded at the beginning of the usability study:

The whole thing stands still. Still. Still doing nothing...The process of downloading the app is pretty clearly signposted, I’m not complaining about that, it just didn’t work.

P2

Technical issues with the Android version also included: lengthy app loading; a blank 7-day weight graph; and the inability to record blood pressure readings, set medication reminders, and use the clock function. Virus-protection interference due to the app being from an unknown source was also reported, regardless of approval of unknown sources in the settings section of the device. The iOS version had less technical issue reports overall but a lengthier multistep initial download process and intermittent screen freezes.

Technical issues were a barrier for ongoing use. Participants commented on the ongoing struggles with the usability:

I’ve persevered with it...but I found I was battling [with the app].

P7

Whether it’s me or whether it’s the program or a combination of both I don’t know, but that’s your problem.

P3

The potential benefit of the app versus the technical challenges associated with the app was also reflected:

I still think the idea is good and I think it’s easy enough to use if it works but I’ve still got problems with the execution, you know.

P2

Interestingly, participants seldom reported technical challenges encountered by the research team during the usability study but raised these issues during the interview.

##### Data Entry

Navigation and data entry were specifically problematic. Participants reported physical limitations during the operation of the app, saying they have “big clumsy fingers” and their “hands shake a little bit” (P7). Participants experienced time-consuming data entry in the medications section, challenges with using some buttons, and confusion completing or updating the settings.

Strategies to overcome these limitations were evident, as participants had insight into their own ailments:

Sometimes I lick the end of my fingers and that might be a factor of fluid, my fluids are very low and I’m quite dry.

P7

Awareness of these functional limitations was a factor in participants choosing a tablet device over a smartphone if they owned both: “I’ve got fat fingers and the phone’s got a small keyboard” (P2). Further, the consequences of incorrect data entry in the settings component of the app caused inappropriate alerts. One participant explained an alert associated with incorrect entry of dry weight:

It told me horror stories about what I should do in terms of consulting my medical practitioners, when in fact I had simply a [settings] error on the machine.

P2

##### Suggested Changes

Many suggested changes were provided in relation to data entry issues, utility by the heart failure population, and making it more appealing for the user.

There were many usability improvements regarding the data entry challenges experienced. Participants wanted more control over their data: “people are generally pretty honest about the way they deal with their own data” (P7). Participants wanted to clear previously entered or incorrect data, edit previously entered data, and enter retrospective data in case it was missed, causing incomplete weight graphs:

If you’re out for the day say and you leave your phone at home and you come back and want to add the data the following day, you can’t do it, so I think that is definitely a negative.

P7

Having an empty data entry screen without predicted or previous amounts was important to avoid confusion during data entries. This was noted for documenting fluid intake and entering daily weight:

It comes up with the last weight you put in so you have to delete that before you can actually [put] a revised weight in and I think that’s a mistake. I think the window should be clear and you just enter in the data you want to enter.

P7

In addition, there were suggestions to improve the applicability to the patient group. These included recording more health data, documenting medication variations more easily, adding a medication checklist function, going over the maximum fluid restriction volume, and adding a free-text general notes page.

Making the user interface more appealing was deemed necessary for engagement with the app. Suggestions included visualization of fluid overflowing out of the fluid jug or turning red in color and more graphical information with an increase to a 14-day trend. Participants explained their wish for a more interesting interface:

If you can have some whistles and bells and things like that–it just makes it a little bit more interesting.

P8

Some screens are very average looking...I think if you could brush it up a little bit and um, make it more appealing some of the screens...would be nice actually.

P6

These improvement suggestions would perceivably improve the utility of the app:

[To] make notes about day to day things…just like a general notes page. That would be a great idea...That would be the decider for me to use it over the other one [app].

P1

Miscellaneous suggested changes included a simpler keyboard, ability to change to horizontal view on the tablet version, and appearance of the logo on more screens.

## Discussion

### Learning from Failure

This paper presents findings from a usability study conducted with patients using an mHealth app for heart failure. We explored the way the app was used and its perceived impact on self-management of disease. In this context, frequently used features were weight and fluid restriction tracking, and graphical representation of data was particularly beneficial. Using technology for self-management would fundamentally differ from current practices; however, use of the app was correlated with the potential utility for daily condition management and as a communication tool. The overall app quality score, as assessed by the MARS, was slightly higher for *Care4myHeart* (3.53) than an average of the 34 comparable heart failure-support apps on the consumer app stores (3.4) [12]. In its current form, the perceived impact on health behavior change was classified as “poor” in the MARS app-specific subscale. Patient experiences of using various app components highlighted challenges and opportunities for design improvements for the next version of the *Care4myHeart* app. In addition, patient experiences have implications for researchers investigating digital health systems for chronic disease and consumer app designers wishing to incorporate human factors. Many lessons were learned from the usability study and are described below.

### Lessons Learned

The following lessons were learned from the evaluation of *Care4myHeart* by patient participants.

#### Lesson 1: If Technology Is Not Integrated Into Everyday Life, It Is a Significant Barrier to Adoption

Integrating self-management with normal life patterns has been identified as a key enabler of effective self-care in heart failure [20], and participants in this study have well-established daily routines. Clarke et al [20] described how patients with heart failure enlist “cues” in everyday life as routines to facilitate guideline adherence. For example, to integrate self-management activities with the morning routine, patients may place pill boxes on the breakfast table as a visual reminder for medication adherence [20]. Participants in the usability study for *Care4myHeart* reported various cues and, except for a few, reported their ease and desire to continue with the existing routines. Demonstrating this, the use of a measuring jug on the kitchen bench for daily fluid restriction management served three functions: a visual reminder to limit oral fluids, a functional measuring tool, and an accurate visual representation of cumulative fluid intake at any point in the day. This presents a more convenient option for participants whose smart devices were located elsewhere in the house and had a more practical option, given the inability of the technology to measure fluid volumes. Participant reflections in comparing the use of technology in heart failure were consistent with the recent study conducted with older people with heart failure: Nguyen et al [9] found that “Some patients did not find technology to be useful or relevant in their daily activities because they were already comfortable with their routines.” Similar reasons likely contributed to the low perceived impact of the app on health behavior change reported in the MARS and indifference to explore all app features, as participants felt the app did not enhance existing self-management. Consequently, introducing the app at the commencement of a self-management regimen may be more beneficial and needs further investigation.

The private nature of smart devices may be a barrier to adoption itself. In this study, no participants reported the involvement of family caregivers regarding the use of the *Care4myHeart* app. Yet, historically, caregivers are frequently involved in heart failure [21] with some patients dependent on their caregivers to make health-related decisions [9]. The gradation of dependency of caregivers for older adults with chronic conditions [22] presents challenges in designing future support interventions [20] when daily health-related activities involve caregivers. The technology risks excluding caregivers unless the design supports their active involvement and the resulting design presents a perceived benefit to the patient *and* caregiver.

#### Lesson 2: The Biggest Benefit Is the Opportunity for Improved Self-Awareness and Continuous Learning in Heart Failure Management

The timely detection and recognition of and action to subtle changes in symptoms was noted as a key skill for effective self-management of heart failure [20]. According to patient experiences, the self-management app we developed offered possibilities for a more active role in daily recording and reviewing of heart failure-related data. Participants specifically observed a benefit in the graphical representation of their data with the ability to view trends, detect changes representative of worsening heart failure, and take action accordingly. Previous studies have shown that skills in managing heart failure evolve over time and learning from past experiences are helpful in applying effective strategies to daily life [21]. This was particularly evident with patients’ experiences using the 7-day weight trend feature. Participants felt it was accurate and timely and provided an objective representation of their health status to watch or act when needed. We believe that the use of mHealth via an app with real-time representation of data trends would strengthen patient empowerment and decision making in self-management.

However, to realize the potential for improved self-awareness and continuous learning, engagement improvements are needed. A recent review, which compared the quality of 34 heart failure support apps on the consumer app stores using the MARS, found the lowest score was for the engagement subscale (2.9/5.0) [12]. This led to a call for further improvements in engagement of mHealth apps for heart failure support. In the context of our study, *Care4myHeart* had an engagement subscale mean of 3.37, which was higher than the average in the review. However, this score still falls short of the “good” range. In this regard, participants conveyed valuable suggestions to improve the interactivity and customization of the app, in addition to suggestions to make the interface more interesting and entertaining. Incorporating the many suggestions provided from (just) six participants in the study may greatly improve the interface for future users. The suggested changes are relatively minor to incorporate in iterations, as they have been in other usability studies [23] achieved through usability studies of similar sample sizes of 5-10 participants [24-26].

#### Lesson 3: Patients Need a Way to Manage Their Health Information Across the Health Care System

The findings of this research indicate that participants want effective ways to share their data with health care professionals for ongoing care. Participants perceived the app to be effective as a communication tool to share their data in a timely, accurate, and visual manner, so that health care professionals can be armed with all relevant health information contained in one system, especially in an emergency or unfamiliar health care setting, for care planning. Australia is transitioning to an opt-out electronic health record; however, during the usability study period, participants’ health information was largely held in silos by individual health providers. Participants reported the safety and quality benefits to record, store, and manage health information in one place, whether it was the *Care4myHeart* app or another assistive technology. These participants’ perspectives are mirrored in a recent study investigating experiences using the patient-accessible electronic health record used in Sweden [27]. Over 96% of survey responders had an overall positive perception of the system, reporting the following highest-rated reasons why they felt it important to have access to their health-related information: (1) it makes patients feel informed, (2) it improves communication between medical staff and the patient, (3) it improves the understanding of the patient’s condition, and (4) it makes patients feel safe [27].

Condition-specific mHealth apps have limitations for integration to current health information systems across acute care, primary care, and community care. Standalone apps will not reach their potential to aid self-management without integration across health care providers, because, like other chronic conditions, patients with heart failure have concurrent comorbid conditions [1], experience frequent hospitalizations [3], and require a team approach across health care sectors [5]. There is increasing recognition that health services for those living with chronic conditions need to be more integrated, coordinated, and patient focused across the continuum of care [2]; however, mHealth has specific challenges in addition to other service redesign efforts. For example, health system readiness, organizational resistance to change, policy uncertainties, and unclear reimbursement schedules for clinicians have been previously identified as barriers to the successful implementation of mHealth technologies for chronic conditions [22].

#### Lesson 4: Technical Challenges are a Significant Barrier to Use With Most Patients Unlikely to Persevere

Attitudes toward technology use impacted participants’ experiences of using the app. The complex components within the app requiring more navigation and data entry, for example, the medication list feature, were infrequently used. These complex components were more likely to have technical and functional issues, which was an additional deterrent reported by participants with less confidence of using technology. For the few participants who self-reported daily app use, the technical challenges were less of a hindrance, but these participants were more likely to provide specific interface-improvement suggestions.

The findings of this usability study have led to recommendations regarding technology use for usability studies conducted with patients, which may be particularly beneficial to clinician researchers. First, testing and re-testing before allowing patients to use the technology is important to help mitigate frustration of poorly functioning technology, a previously reported fear in older adults with heart failure [9]. Second, avoiding version updates during a usability trial will limit confusion, particularly when the researcher cannot screen share with patients located in rural areas to guide the process. Finally, consider recruiting patients who use apps daily as “early adopters” of mHealth for heart failure because of the variable levels of technology acceptance in this patient population [9]. Our findings were consistent with those of Nguyen and colleagues [9] who found that patients were keen to manage their heart failure and willing to uptake self-management recommendations, but discovered that for some patients, adopting a new technology on top of their daily health routines may be of little benefit. Time and effort were barriers to technology acceptance [9], consistent with the findings from this study, where the ease and convenience of continuing with existing self-care regimens outweighed the technical challenges of learning how to use a new app. This would also account for the seldom reporting of technical difficulties during the study. Participants likely made decisions about their acceptance of the app early in the study period and therefore lacked motivation to troubleshoot technical issues with the research team. We found these barriers to technology use regardless of the participant’s keen interest to participate in the research and optimism for technology to assist with their health, noting that the demographic of study participants were older men only.

We tried to minimize technical challenges by using a participatory, co-design approach involving patients in each stage of the development; however, this was not reflected in the study’s findings. This challenges the assumptions of the co-design methodology in addressing the needs of target users and improving usability and places further emphasis on the nonhomogenous attitudes of patients with heart failure when considering technology and health.

### Recommendations for Future Research

Future research should explore in what formats and contexts technology can positively complement daily self-management activities conducted by patients with heart failure. Importantly, we must incorporate the vital caregiver role in the design of condition-specific mHealth because of their active role in self-management support in the home environment. A more focused understanding of the design considerations to engage users in an interesting and beneficial way is likely necessary for adoption and ongoing use, which will require interdisciplinary collaboration between designers, developers, health care providers, and health care consumers. Third-party access to medical information in the app, especially in an emergency, may be an important design recommendation and should be investigated.

With the limited number of evidence-based mHealth interventions moving past the pilot or feasibility stage [22], future studies should investigate the many barriers to adoption and sustainability. Implementation science of mHealth apps for self-management of chronic conditions as an adjunct to existing care is an important area for further research, specifically for investigating perspectives of clinicians, health system administrators, and policy makers.

### Limitations

Since data collection, the authors are aware of a user version of the MARS called uMARS [28], which would have suited this participant sample more specifically as health care consumers. A limitation of this research is the selection bias of the patients. First, as per the inclusion criteria, all participants owned a smart device. Second, less adherent patients, for whom the app may be most beneficial, are often not willing to participate and may have reported different experiences from this sample. The findings from this study conducted with a small and homogenous sample cannot be generalized to the wider heart failure population; nevertheless, they provide insight for further research on the topic.

### Conclusion

A mixed-methods evaluation of patient experiences using an mHealth app for heart failure showed how the app was used and its perceived impact on self-management. Daily self-management habits are established without the use of technology, so patients were unsure how the app would fit in their routines. Nevertheless, participants saw the potential of the app to aid daily condition management, particularly regarding weight and fluid restriction management, and serve as a communication tool for health care professionals involved in their care.

Understanding users’ experiences contributes to design improvements for the *Care4myHeart* app, and the lessons learned have implications for researchers and development teams to advance the quality of consumer mHealth apps for chronic conditions. Future studies should investigate the barriers to adoption and sustainability of consumer mHealth interventions, including whether introducing such apps is more beneficial at the commencement of a self-management regimen. Research into how to incorporate the important role of caregivers in the design of technology to support self-management in the home environment is also needed.

## Appendix

Multimedia Appendix 1 Modified Mobile Application Rating Scale.

## Abbreviations

mHealth: mobile health
MARS: Mobile Application Rating Scale
