# Supplementary material for: Patients’ Experiences of Using a Consumer mHealth App for Self-Management of Heart Failure: Mixed-Methods Study
Source: JMIR Hum Factors. 2019 May 2;6(2):e13009. doi: 10.2196/13009 (PMC6521216; doi:10.2196/13009)
Supplement: Multimedia Appendix 1 [file humanfactors_v6i2e13009_app1.pdf]

# Mobile Application Rating Scale (MARS)

Thank you for taking the time to participate in this research and complete this questionnaire. All items are rated on a 5-point scale from “1. Inadequate” to “5. Excellent”. Please circle the number that most accurately represents the quality of the app component you are rating. Please use the descriptors provided for each response category.

## SECTION A - ENGAGEMENT

**1. Entertainment: Is the app fun/entertaining to use? Does it use any strategies to increase engagement through entertainment (e.g. through gamification)?**

1 Dull, not fun or entertaining at all

2 Mostly boring

3 OK, fun enough to entertain user for a brief time (< 5 minutes)

4 Moderately fun and entertaining, would entertain user for some time (5-10 minutes total)

5 Highly entertaining and fun, would stimulate repeat use

**2. Interest: Is the app interesting to use? Does it use any strategies to increase engagement by presenting its content in an interesting way?**

1 Not interesting at all

2 Mostly uninteresting

3 OK, neither interesting nor uninteresting; would engage user for a brief time (< 5 minutes)

4 Moderately interesting; would engage user for some time (5-10 minutes total)

5 Very interesting, would engage user in repeat use

**3. Customisation: Does it provide/retain all necessary settings/preferences for apps features (e.g. sound, content, notifications, etc.)?**

1 Does not allow any customisation or requires setting to be input every time

2 Allows insufficient customisation limiting functions

3 Allows basic customisation to function adequately

4 Allows numerous options for customisation

5 Allows complete tailoring to the individual’s characteristics/preferences, retains all settings

**4. Interactivity: Does it allow user input, provide feedback, contain prompts (reminders, sharing options, notifications, etc.)? Please note: these functions need to be customisable and not overwhelming in order to be perfect.**

| | |
|---|---|
| 1 | No interactive features and/or no response to user interaction |
| 2 | Insufficient interactivity, or feedback, or user input options, limiting functions |
| 3 | Basic interactive features to function adequately |
| 4 | Offers a variety of interactive features/feedback/user input options |
| 5 | Very high level of responsiveness through interactive features/feedback/user input options |

**5. Target group: Is the app content (visual information, language, design) appropriate for the target audience?**

| | |
|---|---|
| 1 | Completely inappropriate/unclear/confusing |
| 2 | Mostly inappropriate/unclear/confusing |
| 3 | Acceptable but not targeted. May be inappropriate/unclear/confusing |
| 4 | Well-targeted, with negligible issues |
| 5 | Perfectly targeted, no issues found |

| *Office use only* |
|---|
| ***A. Engagement mean score =*** |

# SECTION B - FUNCTIONALITY

**6. Performance: How accurately/fast do the app features (functions) and components (buttons/menus) work?**

| | |
|---|---|
| 1 | App is broken; no/insufficient/inaccurate response (e.g. crashes/bugs/broken features, etc.) |
| 2 | Some functions work, but lagging or contains major technical problems |
| 3 | App works overall. Some technical problems need fixing/Slow at times |
| 4 | Mostly functional with minor/negligible problems |
| 5 | Perfect/timely response; no technical bugs found/contains a ‘loading time left’ indicator |

**7. Ease of use: How easy is it to learn how to use the app; how clear are the menu labels/icons and instructions?**

1 No/limited instructions; menu labels/icons are confusing; complicated

2 Useable after a lot of time/effort

3 Useable after some time/effort

4 Easy to learn how to use the app (or has clear instructions)

5 Able to use app immediately; intuitive; simple

**8. Navigation: Is moving between screens logical/accurate/appropriate/uninterrupted; are all necessary screen links present?**

1 Different sections within the app seem logically disconnected and random/confusing/navigation is difficult

2 Usable after a lot of time/effort

3 Usable after some time/effort

4 Easy to use or missing a negligible link

5 Perfectly logical, easy, clear and intuitive screen flow throughout, or offers shortcuts

**9. Gestural design: Are interactions (taps/swipes/pinches/scrolls) consistent and intuitive across all components/screens?**

1 Completely inconsistent/confusing

2 Often inconsistent/confusing

3 OK with some inconsistencies/confusing elements

4 Mostly consistent/intuitive with negligible problems

5 Perfectly consistent and intuitive

| *Office use only* |
| --- |
| ***B. Functionality mean score =*** |

# SECTION C - AESTHETICS

**10. Layout: Is arrangement and size of buttons/icons/menus/content on the screen appropriate or zoomable if needed?**

1 Very bad design, cluttered, some options impossible to select/locate/see/read device display not optimised

2 Bad design, random, unclear, some options difficult to select/locate/see/read

3 Satisfactory, few problems with selecting/locating/seeing/reading items or with minor screensize problems

4 Mostly clear, able to select/locate/see/read items

5 Professional, simple, clear, orderly, logically organised, device display optimised. Every design component has a purpose

**11. Graphics: How high is the quality/resolution of graphics used for buttons/icons/menus/content?**

1 Graphics appear amateur, very poor visual design - disproportionate, completely stylistically inconsistent

2 Low quality/low resolution graphics; low quality visual design – disproportionate, stylistically inconsistent

3 Moderate quality graphics and visual design (generally consistent in style)

4 High quality/resolution graphics and visual design – mostly proportionate, stylistically consistent

5 Very high quality/resolution graphics and visual design - proportionate, stylistically consistent throughout

**12. Visual appeal: How good does the app look?**

1 No visual appeal, unpleasant to look at, poorly designed, clashing/mismatched colours

2 Little visual appeal – poorly designed, bad use of colour, visually boring

3 Some visual appeal – average, neither pleasant, nor unpleasant

4 High level of visual appeal – seamless graphics – consistent and professionally designed

5 As above + very attractive, memorable, stands out; use of colour enhances app features/menus

| *Office use only* |
|---|
| ***C. Aesthetics mean score =*** |

# SECTION D - INFORMATION

**13. Quality of information: Is app content correct, well written, and relevant to the goal/topic of the app?**

N/A There is no information within the app

1 Irrelevant/inappropriate/incoherent/incorrect

2 Poor. Barely relevant/appropriate/coherent/may be incorrect

3 Moderately relevant/appropriate/coherent/and appears correct

4 Relevant/appropriate/coherent/correct

5 Highly relevant, appropriate, coherent, and correct

**14. Quantity of information: Is the extent coverage within the scope of the app; and comprehensive but concise?**

N/A There is no information within the app

1 Minimal or overwhelming

2 Insufficient or possibly overwhelming

3 OK but not comprehensive or concise

4 Offers a broad range of information, has some gaps or unnecessary detail; or has no links to more information and resources

5 Comprehensive and concise; contains links to more information and resources

**15. Visual information: Is visual explanation of concepts – through charts/graphs/images/videos, etc. – clear, logical, correct?**

N/A There is no visual information within the app (e.g. it only contains audio, or text)

1 Completely unclear/confusing/wrong or necessary but missing

2 Mostly unclear/confusing/wrong

3 OK but often unclear/confusing/wrong

4 Mostly clear/logical/correct with negligible issues

5 Perfectly clear/logical/correct

| *Office use only* |
| --- |
| ***D. Information mean score * =*** |
| **** Exclude questions rated as "N/A" from the mean score calculation.*** |

## SECTION E – QUALITY

**16. Would you recommend this app to people who might benefit from it?**

| | | |
|---|---|---|
| 1 | Not at all | I would not recommend this app to anyone |
| 2 | | There are very few people I would recommend this app to |
| 3 | Maybe | There are several people whom I would recommend it to |
| 4 | | There are many people I would recommend this app to |
| 5 | Definitely | I would recommend this app to everyone |

**17. How many times do you think you would use this app in the next 12 months if it was relevant to you?**

| | |
|---|---|
| 1 | None |
| 2 | 1-2 |
| 3 | 3-10 |
| 4 | 10-50 |
| 5 | >50 |

**18. Would you pay for this app?**

| | |
|---|---|
| 1 | No |
| 3 | Maybe |
| 5 | Yes |

**19. What is your overall star rating of the app?**

| | |
|---|---|
| 1 ★ | One of the worst apps I've used |
| 2 ★★ | |
| 3 ★★★ | Average |
| 4 ★★★★ | |
| 5 ★★★★★ | One of the best apps I've used |

*Office use only*

***Scoring App quality scores for SECTION***
***A: Engagement Mean Score =***
***B: Functionality Mean Score =***
***C: Aesthetics Mean Score =***
***D: Information Mean Score =***
***App quality mean Score =***
***App subjective quality Score =***

# SECTION F – APP SPECIFIC

**20. Awareness: This app is likely to increase my awareness of the importance of addressing heart failure self-care**

1 Strongly disagree

2

3

4

5 Strongly Agree

**21. Knowledge: This app is likely to increase my knowledge/understanding of heart failure self-care**

1 Strongly disagree

2

3

4

5 Strongly Agree

**22. Attitudes: This app is likely to change my attitude toward improving heart failure self-care**

1 Strongly disagree

2

3

4

5 Strongly Agree

**23. Intention to change: This app is likely to increase my intentions/motivation to address heart failure self-care**

1 Strongly disagree

2

3

4

5 Strongly Agree

**24. Help seeking: Use of this app is likely to encourage further help seeking for my heart failure self-care**

1 Strongly disagree

2

3

4

5 Strongly Agree

**25. Behaviour change: Use of this app is likely increase/decrease my heart failure self-care**

1 Strongly disagree

2

3

4

5 Strongly Agree
